# Assessment of the burden on caregivers of patients with mental disorders in Jeddah, Saudi Arabia

**DOI:** 10.1186/s12888-017-1368-1

**Published:** 2017-05-30

**Authors:** Sami H. Alzahrani, Ebtihaj O. Fallata, Marwa A. Alabdulwahab, Wesam A. Alsafi, Jamil Bashawri

**Affiliations:** 1 Family and Community Medicine Department, Faculty of Medicine, King Abdulaziz University, PO Box 80205, Jeddah, 21589 Saudi Arabia; 2 Department of Psychiatry, Mental Health Hospital, Jeddah, Saudi Arabia; 3 Department of Medicine, Ministry of Health, Dammam, Saudi Arabia

**Keywords:** Mentally ill, Caregivers, Burden, Involvement evaluation questionnaire, Saudi Arabia

## Abstract

**Background:**

Mental disorders are considered important public health problems not only to people with mental illness but also their caregivers. As is the case in many countries, the deinstitutionalization of mental health services in Saudi Arabia, has meant that informal caregivers are shouldering responsibilities for which they are not usually prepared; therefore, the current study was aimed at assessment of the burden on caregivers of people with mental illness.

**Methods:**

Through a cross-sectional design, a sample of the caregivers of people with mental illness (*n* = 377) was selected randomly from a psychiatric hospital in Jeddah. An Arabic version of the Involvement Evaluation Questionnaire (IEQ) was used for collection of data. The data were analyzed on the subscale scores and the 27 items in two ways. First, we used the summed scores for the subscales based on the Likert scale (0–4) for univariate and multivariate statistical analyses, as recommended. We also used parametric statistics (t-tests, one-way ANOVA) because the IEQ subscale scores were fairly normally distributed.

**Results:**

Males constituted more than one-half of the participating caregivers (55%), with a mean age of 36.6, SD = 11.4 years. As reported by the caregivers, most of the patients were males (62.7%) with a mean age of 33.8, SD = 13.7 years and a range of 17–90 years old. The total mean IEQ burden score of the caregivers was 38.4, SD = 17.5. “Tension” was significantly prominent among younger caregivers aged ≤30 years. “Worrying” was significantly higher among caregivers living with their spouse and children and those living in families with relatively fewer members (<6 members). “Urging” was significantly higher among caregivers who are living with the patient in the same household and those who had been in close contact with the patient for 28 days in the four weeks prior to the study (13.4, SD = 6.8) *p* < 0.05. Meanwhile, “Urging” was also significantly higher among caregivers caring for mentally ill females (13.5, SD = 6.6) and those not receiving any kind of professional support (12.8, SD = 6.7). The overall burden and the subscale scores were highest among caregivers caring for a close relative such as a parent (44.1, SD = 17.6), son/daughter (39.1, SD = 12.9), sibling (37.1, SD = 18.6), or spouse (37.1, SD = 18.6) *p* < 0.05.

**Conclusion:**

Care for people with mental illness is burdensome for their caregivers, the magnitude of burden is potentially augmented by factors related to the patients and households. These factors should be considered when planning for preparing caregivers to cope with people with mental illness in Saudi Arabia.

## Background

Mental illness is a leading cause of global burden of disease [1]. The burden arises from the distressing nature of mental illness, not only for affected people but also for their family members [2]. Until the mid-1950s, hospitalization of people with mental illness was the routine approach to manage mental illness in many countries. In the latter half of the twentieth century, the process of deinstitutionalization shifted the treatment of these people from state institutions to community care centers [3]. This shift had a substantial impact on the mental health system and the families of people with mental illness because family members were and continue to be often inadequately prepared to be the main caregiver for their ill relatives [4].

Family burden is viewed as the non-mediated effect on families living with and caring for a relative affected by mental illness [5]. There are two defined types of burden: objective burden, which refers to the observable costs to the family that results from the disease; and, subjective burden, which includes the individual’s perception of the situation as burdensome [6]. Specifically, the burden of caring for people with mental illness include disruption of everyday life routine, stigma and blame, dissatisfaction with family and relatives, financial problems, physical burden, troubles with adherence of the patients to treatment and problems with health services and governmental support [7]. It was not until the mid-1950s onwards that the experience of burden of informal family caregivers of family members with mental illness drew researchers’ attention’ [8]. However, while the burden on caregivers has gained considerable attention in Western countries, only a few studies have been conducted in the Middle East to assess the consequences for informal caregivers of caring family members with mental illness. These studies were conducted in Iran [9] and Kuwait [10] and were exclusively focused on caregivers of people with schizophrenia. Saudi Arabia has emphasized mental health as a national priority [11]. The Saudi government has recently passed a Mental Health Act (MHA) that includes support for family members and caregivers for people with mental illness [12]. Services are provided to the patients in the governmental hospitals. The commonest diagnoses include depression, anxiety, psychosis, organic brain syndrome, substance abuse and personality disorders [13]. Nevertheless, research conducted in Saudi Arabia has focused primarily focused on the hospital-based epidemiology of mental disorders and health-services research, leaving a large gap in the field of community mental health services [14]. For these reasons, the current study was aimed to investigating the consequences of caring for family members with mental illness on informal caregivers. In this respect, the Involvement Evaluation Questionnaire (IEQ) was considered appropriate, as it has been used in several other studies as being a valid tool for examining the consequences of caring for people with mental illness on relatives, friends, or other significant persons involved in providing informal, unpaid care [15, 16]. To overcome any concerns that the results could arise from intercultural differences and to facilitate international comparison based on the variation of local standards [15], the questionnaire was translated into Arabic [10] for use in the current study.

## Methods

The caregivers of people with mental illness in a psychiatric hospital in Jeddah were invited to be enrolled in a cross-sectional study that was specially constructed for the study aims. Jeddah is the main seaport of the Kingdom of Saudi Arabia, which, like most of the Gulf countries, has a conservative Muslim culture with traditional gender roles and an extended family system in which family social support is the norm [10]. The respondents (*n* = 377) were selected by systematic random sampling from the attendants of the psychiatric hospital. The caregivers of every second person with mental illness attending the outpatient clinic were invited to participate in the study. To fulfill requirements stated in the inclusion criteria, the cared for person’s mental health diagnosis was confirmed by the treating physician. After approval, participants were asked to complete an Arabic version of the Involvement Evaluation Questionnaire (IEQ), that was used before for collection of data in a similar study [10]. This version was adopted from the IEQ-EU [17]. It is an 81-item instrument that measures the consequences of psychiatric disorders for relatives of people with mental illness in the four weeks preceding the evaluation. Of the 31 items on caregiving consequences, 27 are grouped into four subscales, namely, “Tension” (9 items), “Worrying” (6 items), “Urging” (8 items), and “Supervision” (6 items). In addition, a 27-item total score (summed score for IEQ items 16–35 and 37–43) can be computed. However, two items (IEQ29 on sleep disturbance, and IEQ43 on global subjective rating of burden) are each included in two scales, based on a previous factor analysis report [18]. IEQ29 is included in “Tension” and “Supervision”, while IEQ43 is included in “Tension” and “Worrying”. These items are rated either on a five-point (0–4) Likert scale or a categorical (never/sometimes = 0; and regularly/often/always = 1) scale. The four items not included in the four subscales are IEQ36 (ability to pursue own activities), IEQ44 (getting used to a patient’s problems), IEQ45 (ability to cope with a patient’s problems), and IEQ46 (change in emotional relationship). The total Cronbach’s alpha (internal consistency) of the IEQ items was: 0.93. The subscales internal consistency was: tension: 0.91; supervision: 0.81; worrying: 0.79; urging: 0.89 [10].

We analyzed the data on the subscale scores and the 27 items in two ways. First, we used the summed scores for the subscales based on the Likert scale (0–4) for univariate and multivariate statistical analyses, as recommended [17]. Data were analyzed using SPSS 15 (SPSS Inc., Chicago, Illinois). We used parametric statistics (t-tests, one-way ANOVA) because the IEQ subscale scores were fairly normally distributed.

## Results

Males constituted more than one-half of the participating caregivers (55%), with a mean age of 36.6, SD = 11.4 years, and one-half were married (50.4%) at the time of the study, while the rest were either single, divorced, or widowed. Most of the respondents had either a high school education (40.1%) or university qualifications (31.3%), and the majority of them were living in family contexts, as 41.7% were living with spouses with or without children, and 44.1% were living with close family members. Additionally, more than one-half of the caregivers (55%) indicated that there were six or more family members in the same household. Two-thirds of the caregivers (66.5%) had been living with the family members with mental illness in the same household for four weeks prior to the study (64.9%). The great majority of the caregivers (82%) had family income <7500 SR (2000 USD) [Table 1]. As reported by the caregivers, most people with mental illness were males (62.7%) 17–90 years of age with a mean age of 33.8, SD = 13.7 years. The majority of the people with mental illness were related to their caregivers in some way: parents (20.2%), sons/daughters (15.3%), siblings (40.9%), or husband/wife (8.2%). The caregivers stated that 16.9% of the family members with mental illness were not receiving any kind of support, while those who had support were receiving it mainly from the psychiatric hospital, either in the outpatient clinic (51%) or as a registered patient in the hospital (48.5%) [Table 2].

**Table 1:** Characteristics of the caregivers (*n* = 367)

Characteristics	No.	Percent
Gender		
Male	202	55.0
Female	165	45.0
Age groups		
≤ 30 years	128	34.9
31–40 years	133	36.2
≥ 41 years	106	28.9
Range	20–85 years	
Mean (SD)	36.6 (11.4) years	
Marital status		
Single	118	32.2
Married	185	50.4
Divorced	44	12.0
Widowed	20	5.4
Education level		
Illiterate	30	8.2
Primary	25	6.8
Intermediate	50	13.6
High school	147	40.1
University	115	31.3
Living arrangement		
Lives alone	25	6.8
Lives with spouse/children	153	41.7
Lives with family siblings	162	44.1
Lives with other relatives	12	3.3
Lives with friends	15	4.1
Number of family members in the same household		
< 6	165	45.0
6–9	146	39.8
> 9	56	15.3
Living with the person with mental illness in the same household		
Yes	244	66.5
No	123	33.5
Days of contact with the person with mental illness in the past four weeks		
No contact	51	13.9
Contact for 1- < 28 days	78	21.2
Contact for 28 days	238	64.9
Monthly income in SR: SR = 0.267 US $		
less than 1500	48	13.0
1500- < 2500	85	23.2
2500- < 4500	91	24.8
4500- < 7500	77	21.0
7500–11,250	36	9.8
more than 11,250	30	8.2

**Table 2:** Characteristics of the people with mental illness

Characteristics	No.	Percent
Gender		
Male	230	62.7
Female	137	37.3
Age groups		
≤ 20 years	31	8.4
21–30 years	158	43.1
31–40 years	100	27.2
41+ years	78	21.3
Range	17–90 years	
Mean (SD)	33.8 (13.7) years	
Relationship with the caregivers		
Parents	74	20.2
Sons/daughters	56	15.3
Siblings	150	40.9
Other relatives	20	5.4
Husband/wife	30	8.2
Friend	29	7.8
Neighbor	8	2.2
Type of support		
No support	62	16.9
Support from GP or family physician	16	4.4
Support from social specialist	30	8.2
Support from psychiatrist	87	23.7
Support from outpatient clinics in psychiatric hospital	187	51.0
Support as being a registered patient in psychiatric hospital	178	48.5

The total mean IEQ burden score of the caregivers was 38.4, SD = 17.5. The scores of the four IEQ sub-scales showed marked variance, with “Worrying” as the most highly affected, with an average score of (1.8, SD = 0.9), followed by “Urging” (1.6, SD = 0.8). The least affected were “Tension” (1.2, SD = 0.9) and “Supervision” (1.2, SD = 0.8) [Table 3]. “Tension” was significantly prominent among younger caregivers, where the average ranged between (11.0, SD = 8.2) and (11.5, SD = 7.7) for caregivers aged ≤30 years and 31–40 years, respectively, compared to (8.8, SD = 7.8) for caregivers aged ≥41 years. “Worrying” was highest among caregivers living with their spouse and children (11.7, SD = 5.3) and those living in families with relatively fewer members (<6 members) (11.5, SD = 6.0). Meanwhile, “Urging” was highest among caregivers living with the family members with mental illness in the same household and those who had been in close contact with the them for 28 days over the four weeks prior to the study (13.4, SD = 6.8) *p* < 0.05. Otherwise, no other characteristics of the caregivers were found to significantly affect their perceived burden (*p* > 0.05) [Table 4].

**Table 3:** Average scores of the IEQ burden subscale

IEQ subscales	Mean, SD*	Possible range*	Actual range	Mean, SD**
Tension (9 items)	10.6, SD = 8.0	0–36	0–36	1.2, SD = 0.9
Supervision (6 items)	7.5, SD = 5.4	0–24	0–24	1.2, SD = 0.8
Worrying (6 items)	10.7, SD = 5.6	0–24	0–24	1.8, SD = 0.9
Urging (8 items)	12.5, SD = 6.6	0–32	0–32	1.6, SD = 0.8
Overall score (27 items)***	38.4, SD = 17.5	0–108	0–108	

*Based on sum of the Likert scale response options: 0–4 (Van Wijingaarden et al. 2000)

**Based on average of the Likert scale response options: 0–4

***IEQ29 is included in “tension” and “supervision; IEQ43 is included in “tension” and “worrying”

**Table 4:** Differences in the IEQ subscale scores according to characteristics of the caregivers

Statements	Burden subscales				Overall score Mean, SD
	Tension Mean, SD	Supervision Mean, SD	Worrying Mean, SD	Urge Mean, SD	
Gender					
Male	11.2, SD = 8.5	8.0, SD = 5.9	10.6, SD = 5.6	12.3, SD = 6.7	39.0, SD = 18.7
Female	9.7, SD = 7.4	6.9, SD = 4.7	10.9, SD = 5.6	12.8, SD = 6.4	37.8, SD = 16.1
*P*	0.083	0.063	0.655	0.484	0.531
Age					
≤ 30 years	11.0, SD = 8.2	7.8, SD = 5.5	11.3, SD = 5.2	11.7, SD = 6.2	38.9, SD = 16.6
31–40 years	11.5, SD = 7.7	7.9, SD = 5.3	10.4, SD = 5.3	13.1, SD = 6.6	39.9, SD = 17.9
≥41 years	8.8, SD = 7.8	6.5, SD = 5.3	10.6, SD = 6.4	12.9, SD = 7.0	36.0, SD = 18.2
*P*	0.034	0.126	0.439	0.192	0.264
Marital status					
Single	10.9, SD = 8.6	7.4, SD = 5.7	10.2, SD = 5.3	11.7, SD = 7.1	37.4, SD = 18.7
Married	10.6, SD = 7.7	7.6, SD = 5.3	11.2, SD = 5.8	13.3, SD = 6.3	39.7, SD = 16.8
Divorced	9.6, SD = 8.1	7.0, SD = 5.1	10.6, SD = 5.7	11.3, SD = 6.7	36.1, SD = 18.7
Widowed	10.3, SD = 7.7	7.6, SD = 5.0	10.4, SD = 4.8	12.8, SD = 5.9	38.2, SD = 14.4
*P*	0.852	0.919	0.490	0.138	0.574
Education level					
Illiterate	11.3, SD = 9.6	7.1, SD = 5.9	10.3, SD = 6.6	14.7, SD = 7.4	40.4, SD = 20.5
Primary	12.5, SD = 8.9	9.9, SD = 5.5	11.6, SD = 5.9	14.2, SD = 5.2	43.7, SD = 17.6
Intermediate	9.8, SD = 6.8	7.4, SD = 5.0	11.1, SD = 5.1	12.3, SD = 5.7	38.3, SD = 14.8
High school	10.6, SD = 8.0	7.3, SD = 5.2	10.2, SD = 5.4	11.5, SD = 6.7	36.9, SD = 17.5
University	10.2, SD = 8.0	7.4, SD = 5.5	11.2, SD = 5.6	12.9, SD = 6.7	38.8, SD = 17.7
*P*	0.720	0.330	0.516	0.092	0.509
Living arrangement					
Live alone	13.4, SD = 8.3	8.1, SD = 4.7	9.0, SD = 5.4	14.1, SD = 7.7	41.0, SD = 18.4
Live with spouse/children	10.1, SD = 7.2	7.0, SD = 4.7	11.7, SD = 5.3	13.4, SD = 6.0	39.4, SD = 15.0
Lives with family siblings	10.9, SD = 8.9	7.9, SD = 6.1	10.4, SD = 5.8	11.6, SD = 6.9	37.7, SD = 19.8
Lives with other relatives	8.8, SD = 5.1	7.1, SD = 3.6	9.0, SD = 4.4	13.5, SD = 6.8	36.6, SD = 14.6
Lives with friends	8.9, SD = 6.0	7.3, SD = 5.4	9.1, SD = 5.7	10.7, SD = 5.4	34.1, SD = 16.6
*P*	0.331	0.670	0.038	0.072	0.717
Number of family members					
<6 members	10.6, SD = 7.9	7.4, SD = 5.0	11.5, SD = 6.0	12.9, SD = 6.7	39.8, SD = 17.4
6–9 members	10.0, SD = 8.2	7.4, SD = 5.8	10.2, SD = 5.2	12.6, SD = 6.6	37.2, SD = 17.0
>9 members	11.7, SD = 7.8	7.8, SD = 5.6	9.7, SD = 5.9	11.7, SD = 6.2	37.8, SD = 19.3
*P*	0.410	0.915	0.045	0.547	0.454
Live with person with mental illness in same household					
Yes	10.2, SD = 8.4	7.5, SD = 5.5	11.0, SD = 5.7	13.2, SD = 6.7	38.9, SD = 17.7
No	11.4, SD = 7.2	7.5, SD = 5.2	10.3, SD = 5.2	11.0, SD = 6.0	37.5, SD = 17.3
*P*	0.207	0.883	0.242	0.004	0.507
Days of contact					
No contact	8.9, SD = 7.1	6.5, SD = 5.1	10.7, SD = 6.0	11.3, SD = 7.0	34.8, SD = 20.3
Contact 1- < 28 days	11.1, SD = 7.0	7.4, SD = 4.4	9.7, SD = 5.1	10.4, SD = 4.9	35.7, SD = 13.8
28 days	10.6, SD = 8.4	7.7, SD = 5.7	11.1, SD = 5.6	13.4, SD = 6.8	39.9, SD = 18.1
*P*	0.409	0.504	0.145	0.001	0.091
Monthly income					
< 1500 SR	11.1, SD = 8.7	8.4, SD = 6.2	9.3, SD = 5.8	12.6, SD = 7.4	38.4, SD = 21.6
1500- < 2500	10.9, SD = 7.9	7.3, SD = 5.3	9.9, SD = 5.8	11.7, SD = 6.2	37.1, SD = 16.6
2500- < 4500	9.9, SD = 7.7	7.0, SD = 5.1	11.1, SD = 5.6	13.0, SD = 6.9	38.3, SD = 17.6
4500- < 7500	10.5, SD = 7.7	7.4, SD = 4.3	11.2, SD = 4.8	11.9, SD = 5.6	37.7, SD = 13.5
7500–11,250	11.1, SD = 7.5	8.4, SD = 6.6	11.9, SD = 5.5	14.3, SD = 6.4	42.7, SD = 18.4
more than 11,250	10.4, SD = 7.8	7.0, SD = 5.5	11.8, SD = 6.2	12.3, SD = 7.3	39.1, SD = 20.5
*P*	0.946	0.668	0.143	0.430	0.776

Regarding patients’ characteristics, “Urging” was significantly higher among caregivers of females with mental illness (13.5, SD = 6.6) and those not receiving any kind of professional support (i.e. family physician, psychiatrist, social worker and psychiatry home care programs) (12.8, SD = 6.7). The overall burden, as well as the individual subscale scores, were significantly affected by the relationship between the family members with mental illness and their caregivers. The highest score of burden was observed among caregivers of close relatives such as parents (44.1, SD = 17.6), sons/daughters (39.1, SD = 12.9), siblings (37.1, SD = 18.6), and spouses (37.1, SD = 18.6) *p* < 0.05 [Table 5].

**Table 5:** Differences in the IEQ subscale scores according to characteristics of the people with mental illness

Statements	Burden subscales				Overall score Mean, SD
	Tension Mean, SD	Supervision Mean, SD	Worrying Mean, SD	Urge Mean, SD	
Gender					
Male	10.9, SD = 8.2	7.6, SD = 5.4	10.8, SD = 5.5	11.9, SD = 6.5	38.5, SD = 17.8
Female	9.9, SD = 7.7	7.2, SD = 5.3	10.6, SD = 5.7	13.5, SD = 6.6	38.3, SD = 17.1
*P*	0.250	0.497	0.774	0.033	0.926
Age					
< 21 years	9.0, SD = 7.5	7.6, SD = 6.3	11.7, SD = 6.2	14.1, SD = 6.4	40.0, SD = 17.3
21–30 years	10.4, SD = 7.4	7.5, SD = 5.0	11.1, SD = 5.3	12.3, SD = 6.6	38.3, SD = 16.1
31–40 years	11.1, SD = 7.5	7.7, SD = 5.2	9.9, SD = 5.4	11.9, SD = 6.3	38.1, SD = 17.9
≥41 years	10.9, SD = 7.8	6.9, SD = 5.9	10.8, SD = 6.1	13.2, SD = 7.0	38.3, SD = 20.6
*P*	0.633	0.827	0.300	0.338	0.965
Relationship with caregiver					
Parents	13.2, SD = 9.7	8.4, SD = 5.7	11.7, SD = 5.5	14.6, SD = 5.8	44.1, SD = 17.6
Sons/daughters	8.5, SD = 7.2	6.7, SD = 5.0	12.6, SD = 5.4	13.7, SD = 6.2	39.1, SD = 12.9
Siblings	10.9, SD = 7.5	7.7, SD = 5.4	10.4, SD = 5.7	11.1, SD = 6.6	37.1, SD = 18.6
Other relatives	8.5, SD = 8.9	7.0, SD = 5.6	8.5, SD = 5.7	10.8, SD = 5.5	32.3, SD = 19.2
Spouse	8.8, SD = 7.2	6.7, SD = 4.9	9.8, SD = 4.9	14.3, SD = 7.8	36.2, SD = 18.9
Friend	8.8, SD = 6.6	6.7, SD = 5.0	8.8, SD = 5.4	11.5, SD = 6.4	33.5, SD = 15.7
Neighbor	9.7, SD = 6.1	7.2, SD = 6.8	11.7, SD = 2.8	10.7, SD = 67.6	36.8, SD = 14.6
*P*	0.011	0.458	0.011	0.003	0.036
Receiving professional support					
Yes	10.8, SD = 8.6	7.1, SD = 5.4	11.9, SD = 5.4	11.0, SD = 5.6	36.8, SD = 17.0
No	10.5, SD = 7.9	7.6, SD = 5.4	10.5, SD = 5.6	12.8, SD = 6.7	38.7, SD = 17.6
*P*	0.769	0.554	0.078	0.050	0.476

## Discussion

Family burden has become an essential indicator for mental health service provision; however, differences in social networks and culture could lead to variation in the experience of burden among caregivers of people with mental illness in different countries [5]. For example, in Arab countries in general, religious culture is most prevalent, and, among extended family, there is a tradition of rallying around those family members who are ill [10]. This could explain the differences in the overall IEQ burden score (38.4, SD = 17.5) observed among our caregivers as compared to the pooled mean scores from five European countries (50.6, SD = 16.3), where the highest mean score was reported in Verona (Italy) (56.6, SD = 18.6) and the lowest in Copenhagen (Denmark) (41.3, SD = 9.4) [17]. In a study done in Portugal, a much lower score was reported (30.2, SD = 14.7) [19]. Thus, any interpretation and comparability of the results from the current study with previous European reports should be considered with caution due to differences in social norms in addition to the extent of the services performed to meet each patient’s needs [10].

Regarding the age of the caregivers as a predictor for the magnitude of burden experienced while caring for people with mental illness, the reviewed literature revealed diverse findings; for example, while Juvang found that older caregivers of people with mental illness in China experienced increased burden [20], the reverse was found in the case of Mexican caregivers, where the younger caregivers felt the highest level of burden [21]. In our study, “Tension” was significantly prominent among younger caregivers, where the average ranged between (11.0, SD = 8.2) and (11.5, SD = 7.7) for caregivers aged ≤30 years and 31–40 years, respectively, compared to (8.8, SD = 7.8) for caregivers aged ≥41 years. The relatively lower burden among older caregivers could be explained by the acquired cumulative experience in dealing with illness and crisis among people with mental illness [22].

It was initially hypothesized that not only do caregivers’ characteristics affect their psychological state and the degree of the burden they experience, but the characteristics of the people with mental illness contribute to caregivers’ distress as well. In the current study, in contrast to the report from the EPSILON study that there was no significant difference by people with mental illness gender [23], our findings revealed that caring for female with mental illness significantly increases the “Urging” burden on caregivers. This burden was significantly higher among caregivers of females with mental illness, which is a fairly uncommon finding: few studies have reported that this burden was associated with caring for females [24]. This difference could be explained by the variation in cultural norms between Western and Arab communities. In this respect, Zahid and Othaeri stated that “issues related to women are handled with secrecy in the Arab culture, and it would be relatively more distressing if the females with mental illness behavior remained disruptive, especially as this would curtail her chances of marriage in a culture where traditionally arranged marriage is the order of the day” [10]. Another explanation was given by Winefield and Harvey, who referred to the gender difference of the people with mental illness as a determinant of the extent of burden on their caregivers as due to the differences in behavioral expectations for the two sexes. They also stated that perhaps “the effect of ill women on their children caused greater anxiety” as females are more likely to be incapacitated in caring for their children [24].

The overall burden, as well as the subscale scores, were significantly affected by the relationship between the people with mental illness and their caregivers. The highest score of burden was observed among caregivers caring for close relatives such as parents, sons/daughters, siblings, and spouses. This could be attributed to feelings of grief and sorrow for the family member [25], disrupted social relationships and isolation [26], and the consequent disturbances in daily activities required to fulfill the special care needs of the people with mental illness. This experienced burden among studied caregivers, as expected, was particularly apparent among caregivers who usually live with the cared for person in the same household. These findings are supported by previous studies which indicated that higher burden was associated with an increased number of hours spent caring for the family members with mental illness [18, 27]. When the responsibilities of caring for the family members with mental illness are distributed over more than one member of the family, the burden is expected to be much less [28–30]. Accordingly, in the current study, “Worrying” was highest among caregivers living in families with relatively fewer members (<6 members).

Even after deinstitutionalization of caring for people with mental illness, the role of professional support cannot be overlooked. One role of professionals is to prepare caregivers for dealing with caring for their relatives with mental illness [31]. This notion was evident among our caregivers, where the burden of “Urging” was significantly higher among those who reported a lack of professional support.

## Conclusion and recommendations

Care for people with mental illness is burdensome for their caregivers, the magnitude of burden is potentially augmented by factors related to the patients and households such as being a young patient, living with fewer family members, and factors related to the caregivers such as being a female closely related to the patients (mother, daughter or spouse). These factors should be considered when planning for preparing caregivers to cope with people with mental illness in Saudi Arabia.

## Limitations of the study

The major limitations of the study are that it was cross-sectional, and therefore participants could not be considered as representative of all informal caregivers of people with mental illness in Saudi Arabia. Additionally, the impact of that only one of the caregivers who was in companion with the patients was interviewed. However, the use of an internationally validated questionnaire made our findings comparable with the international reports.

## Abbreviations

IEQ: Involvement Evaluation Questionnaire
SPSS: Statistical Package for the Social Sciences
